# High Prevalence of Hepatitis B Virus Infection Among People Living with Advanced HIV Disease in Botswana †

**DOI:** 10.3390/biomedicines14061229

**Published:** 2026-05-29

**Authors:** Chanana D. Tsayang, Emily Schanzer, Bonolo B. Phinius, Graceful Mulenga, Kesaobaka Molebatsi, Kwana Lechiile, Lynnette Bhebhe, Tsholofelo Ratsoma, Gorata G. A. Mpebe, Fredah Mulenga, Basetsana K. S. Phakedi, Wonderful T. Choga, Madisa Mine, Shahin Lockman, Joseph N. Jarvis, Sikhulile Moyo, Motswedi Anderson, Simani Gaseitsiwe

**Affiliations:** 1 Botswana Harvard Health Partnership, Gaborone Private Bag BO 320, Botswana; ctsayang@bhp.org.bw (C.D.T.); emilyschanzer@college.harvard.edu (E.S.); bphinius@bhp.org.bw (B.B.P.); gmulenga@bhp.org.bw (G.M.); kmolebatsi@bhp.org.bw (K.M.); lechiilek@hotmail.com (K.L.); lynnettebhebhe@gmail.com (L.B.); tratsoma@bhp.org.bw (T.R.); gmpebe@bhp.org.bw (G.G.A.M.); fmulenga@bhp.org.bw (F.M.); bphakedi@bhp.org.bw (B.K.S.P.); wchoga@bhp.org.bw (W.T.C.); slockman@hsph.harvard.edu (S.L.); drjoejarvis@gmail.com (J.N.J.); smoyo@bhp.org.bw (S.M.); manderson@bhp.org.bw (M.A.); 2 Department of Mathematics and Statistical Sciences, Faculty of Science, Botswana International University of Science and Technology, Palapye Private Bag 16, Botswana; 3 Department of Biological Sciences, Faculty of Science, University of Botswana, Gaborone Private Bag 00704, Botswana; 4 School of Allied Health Professions, Faculty of Health Sciences, University of Botswana, Gaborone Private Bag 0022, Botswana; 5 Ministry of Health, Gaborone Private Bag 0038, Botswana; madisamine0@gmail.com; 6 Department of Immunology and Infectious Diseases, Harvard T. H. Chan School of Public Health, Boston, MA 02115, USA; 7 Department of Clinical Research, Faculty of Infectious and Tropical Diseases, London School of Hygiene and Tropical Medicine, London WC1E 7HT, UK; 8 Division of Medical Virology, Faculty of Medicine and Health Sciences, Stellenbosch University, Cape Town 7505, South Africa; 9 School of Health Systems and Public Health, University of Pretoria, Pretoria 0002, South Africa; 10 Africa Health Research Institute, Durban 4013, South Africa; 11 The Francis Crick Institute, London NW1 1AT, UK

**Keywords:** advanced HIV disease, hepatitis B virus, people living with HIV, adjusted prevalence ratios, immunocompromised

## Abstract

**Background**: Concomitant HIV/HBV infection results in worse health outcomes, with HBV reactivations being observed in immunocompromised individuals. However, data on HBV infection in people with advanced HIV disease (AHD) remains sparse in Botswana. We aimed to determine the prevalence and molecular characteristics of HBV in people living with HIV (PLHIV) with CD4+ T-cell counts ≤100 cells/µL in Botswana. **Methods**: Plasma samples (*n* = 1097) of PLHIV with CD4+ T-cell count ≤100 cells/uL collected between 2014 and 2016 were screened for hepatitis B surface antigen (HBsAg) and HBV core antibodies (anti-HBc). A 415bp region of the HBV surface gene was amplified and sequenced using Sanger sequencing. Genotypic and mutational analysis was performed using Geno2pheno. Adjusted prevalence ratios (aPRs) were estimated from a modified Poisson regression model to explore factors associated with HBV infection. *p*-values < 0.05 indicated statistical significance. **Results**: The median age was 37 years (IQR: 32–43), and 565/1097 (51.5%) were male. HBsAg prevalence was 10.6% (95%CI: 8.8–12.5%) and anti-HBc prevalence was 50.0% (95%CI:46.9–52.9%). Factors associated with HBV infection were male sex [aPR: 1.6 (*p* < 0.01)] and those that were ART-experienced [aPR: 1.43 (*p* = 0.04). Eighteen samples were successfully genotyped. The prevalence of genotype A was (12/18, 66.7%) and D (6/18, 33.3%). Sixty-three mutations were identified as associated with drug resistance and immune and diagnostic escape. Highly prevalent immune escape mutations in the surface region were S207N (12/63, 19%) and A194V (9/63, 14.3%). V163I (12/63, 19%) and M129L (12/63, 19%) were highly prevalent in the reverse transcriptase region. Two classical lamivudine-associated drug resistance mutations were observed, each occurring in one participant (L180M and V173L). **Conclusions**: The prevalence of HBV in people with AHD is high, highlighting the importance of HBV screening and HIV/HBV co-management in this population.

## 1. Introduction

Hepatitis B virus (HBV) and human immunodeficiency virus (HIV) remain major global health challenges. In 2022, an estimated 254 million individuals were living with chronic hepatitis B (CHB). According to the World Health Organization (WHO) in 2023, there were approximately 1.5 million new HBV infections annually. Roughly 1 million deaths annually are due to end-stage liver disease (ESLD) or hepatocellular carcinoma (HCC) resulting from HBV infection. Approximately 39.0 million people were living with HIV (PLHIV) at the end of 2022. It is estimated that 2.73 million PLHIV worldwide are co-infected with HBV [1].

Concomitant HBV/HIV results in rapid progression of both diseases. In PLHIV, HBV infection is more likely to become chronic [2,3]. Additionally, people with HIV/HBV tend to have fewer CD4+ T lymphocytes and a slower recovery of these cells compared to those with HIV mono-infection [2,4]. They also show a poorer virologic response when undergoing combination antiretroviral therapy (cART) as compared to those with HBV mono-infection [5].

In Botswana, the adult HIV-1C prevalence is reported to be ~16–20%, while the HBsAg prevalence in PLHIV ranges between 1.7 and 10.6% in adults [6,7] and the prevalence of hepatitis B core antibodies (anti-HBc) is 56% [8]. HBV genotypes A, D, and E have been previously reported in Botswana [9]. The progression and natural course of HBV varies across different genotypes/sub-genotypes, influencing both clinical and treatment strategies [10,11].

Immunocompromised individuals due to immunosuppressive therapy or HIV co-infection are at high risk of HBV reactivation [12,13]. HBV reactivation can lead to progressive HBV outcomes such as increased risk of liver cirrhosis and HCC, leading to high morbidity and mortality [13,14]. However, data on HBV infection in people with advanced HIV disease (AHD) remains sparse in Botswana. Most of the studies that have been done in Botswana previously have mainly focused on PLHIV with a CD4+ T-cell count of >200 cells/µL who are deemed to be relatively immune-competent. This study aimed to determine the prevalence of HBV and molecular characteristics of HBV among people with AHD with CD4+ T-cell counts ≤100 cells/µL in Botswana who are in the category of immunosuppressed.

## 2. Materials and Methods

### 2.1. Study Population and Ethical Approval

This retrospective cross-sectional study utilized stored plasma samples of a cohort used to investigate cryptococcal antigen (CrAg) positivity at different CD4+ T-cell counts. The study enrolled PLHIV with CD4+ T-cell counts ≤100 cells/µL, defined as advanced HIV disease (AHD) from 2014 to 2016 in greater Gaborone. Of the 2108 participants, 1097 plasma samples had sufficient volumes and were retrieved. Ethical approval for this study was granted by the Human Research Development Committee at the Botswana Ministry of Health (HPRD 6/14/1).

### 2.2. HBV Serological Assays

Plasma samples were screened for the HBsAg using Murex HBsAg version 3. An Enzyme-linked Immunosorbent assay (ELISA) kit was used (Murex Biotech, Dartford, UK), with all HBsAg-positive results confirmed by a repeat run of the same assay. Samples were also screened for total anti-HBc using the MONOLISA Anti-HBc PLUS (Bio-Rad, Marnes-la-Coquette, Paris, France).

### 2.3. HBV Surface Gene Amplification

DNA was extracted from 200 µL of plasma using the Qiagen DNA Blood mini kit (Qiagen, Hilden, Germany) with a modified final elution volume of 30 µL. The protocol was adopted from [9]. A 415 bp fragment of the HBV surface gene was amplified using the Taq polymerase enzyme. A semi-nested polymerase chain reaction (PCR) was performed with a total reaction volume of 50 μL. The first-round PCR conditions were as follows: initial denaturation at 94 °C for 5 min, followed by 30 cycles of denaturation at 94 °C for 45 s, annealing at 50 °C for 30 s, and elongation at 72 °C for 90 s, with a final extension at 72 °C for 10 min. The primers used in the first-round PCR were HBV381 (5′-TGC GGC GTT TTA TCA TCT TCC T-3′, position 381–402) and HBV840 (5′-GTT TAA ATG TAT ACC CAA AGA C-3′, position 840–861) [15]. The second PCR round began with a 5 min denaturation at 94 °C, followed by 30 cycles of denaturation at 94 °C for 45 s, annealing at 55 °C for 30 s, and elongation at 72 °C for 60 s, concluding with a final extension at 72 °C for 10 min. Primers used for the PCR round were HBV381 and HBV801 (5′-CAG CGG CAT AAA GGG ACT CAA G-3′, position 801–822) [15]. The amplified samples were visualized on a 1% agarose gel stained with ethidium bromide to confirm successful amplification.

### 2.4. Sequencing and Bioinformatics Analysis

Amplified PCR products were purified using Exo-SAP (Applied Biosystems, Vilnius, Lithuania) for cleanup according to the manufacturer’s instructions. Sequencing was performed using BigDye sequencing chemistry on an ABI 3500xl genetic analyzer (Thermo Fisher Scientific, Waltham, MA, USA). For downstream analysis, Sequencher (Sequencher, Ann Arbor, MI, USA) was used to clean up raw chromatogram data and to generate consensus sequences using overlapping sequences. The consensus sequences were viewed as FASTA files in Aliview, where editing and alignment of sequences was done. Geno2Pheno (https://www.geno2pheno.org, accessed on 26 August 2024) was used for genotypic and mutational analysis. Botswana reference HBV sequences used for phylogenetic analysis were obtained from NCBI. The phylogenetic analysis was done using Bayesian Evolutionary Analysis with the Sampling Trees software (BEAST 1.7), including reference Botswana sequences and sequences generated from the study. The software constructed the tree using a statistical method called Markov chain Monte Carlo (MCMC) to investigate different phylogenetic models [16]. The MCMC was run for 10,000,000 times, with parameters logged every 1000. The tree was then visualized using FigTree v1.4.3.

### 2.5. Statistical Analysis

Participants’ clinical and demographics characteristics were summarized in frequencies and percentages and medians with interquartile ranges (IQRs). Fisher’s exact test was used for categorical data and the chi squared test was used for continuous variables. These statistical analyses were conducted using Stata version 18.0 (Stata Corp LLC, College Station, TX, USA) and *p*-values < 0.05 were deemed statistically significant. Prevalence ratios for HBV positivity were determined using the modified Poisson regression model. Variables with *p*-values of 0.2 or less in the univariate model were included in the multivariate Poisson regression analysis, where a *p*-value of less than *p* < 0.05 was statistically significant. We used Multiple imputation by chain equations (MICE) to account for missing viral loads. Missingness was assumed to be Missing at Random (MAR). This assumption was considered because the main factors related to viral load recording, including ART status and CD4 count, were fully observed and included in the imputation model. Imputation was performed using the mice package in R v4.4.2. Five imputed datasets were generated using predictive mean matching. Results from the five datasets were combined using Rubin’s rules. The imputed viral suppression variable was then included in both univariate and multivariable Poisson regression analysis. Data obtained was analyzed using R version R-4.4.2 (RStudio, Inc., Boston, MA, USA; 2021).

## 3. Results

### 3.1. Participants Characteristics and Risk Factors

The CrAg study enrolled 2108 participants, of which 1097 (52.04%) samples were available for the current analysis. A total of 1097 samples were screened for HBsAg and 1082 for anti-HBc. The prevalence of HBsAg + was 10.6% (95% CI: 8.8–12.5%), indicating that 116 participants tested positive, and anti-HBc prevalence was 50.0% (95% CI: 46.9–52.9%), showing that 541 of the participants tested positive for anti-HBc. HBsAg+/anti HBc- prevalence was 15/112 (13.4%).

The median age of participants was 37 years (IQR: 32–43), with most of the participants being males, 565/1097 (51.5%), as shown in Table 1. For ART status, 571/1097 (52.1%) were naïve, 526/1097 (47.9%) participants were ART-experienced and the median CD4+ T-cell count was 54 cells/uL (IQR: 27–77). In the univariate analysis, being male [PR:1.59 (*p* = 0.015)] and ART-experienced [PR: 1.43 (*p* = 0.04)] was associated with HBsAg infection, as shown in Table 2. After adjusting for sex and ART status, both male sex and ART status were associated with HBsAg positivity [aPR: 1.6 (*p* < 0.01)]. CrAg positivity was found in 52 (5.3%) HBsAg- and five (4.3%) HBsAg+ participants, with no statistically significant association between the two (*p* = 0.6).

### 3.2. Phylogenetic Tree

**Figure 1:**
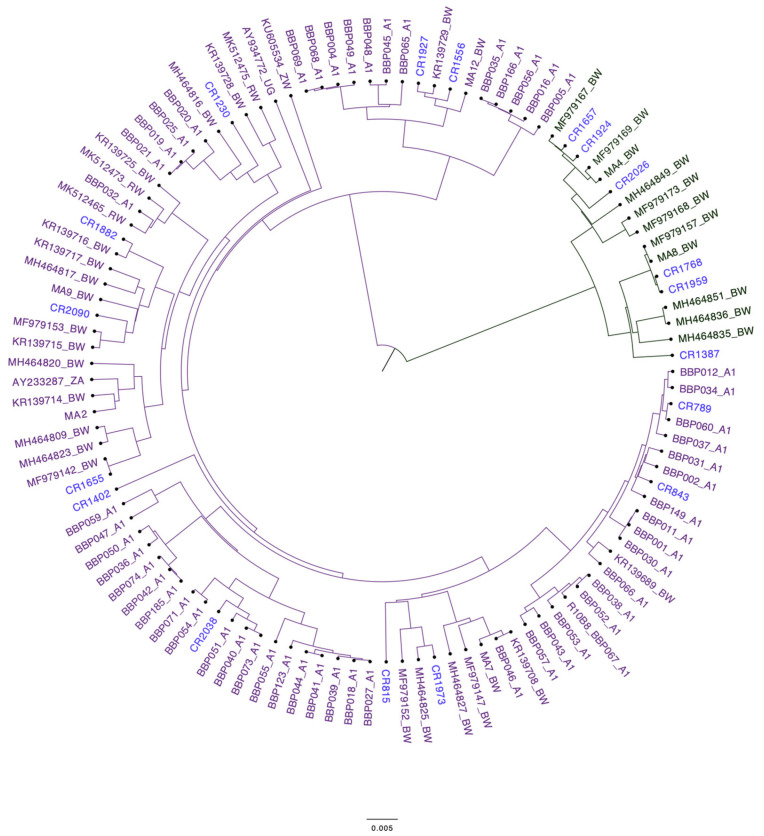
Phylogenetic tree of people with AHD in Botswana, sequences generated from study in blue) and reference HBV sequences from Botswana (in purple for genotype A and green for genotype D).

Sequencing was successful in 18 participants owing largely to insufficient sample volumes. The phylogenetic tree of 18 HBV sequences from people with AHD was constructed using BEAST. The phylogenetic analysis revealed that the sequences were genotype A in purple [12/18, 66.7%] and genotype D in green [6/18, 33.3%]. Genotypes A and D of our generated sequences clustered with previous HBV sequences from Botswana, as shown in Figure 1 below.

### 3.3. Mutations

**Figure 2:**
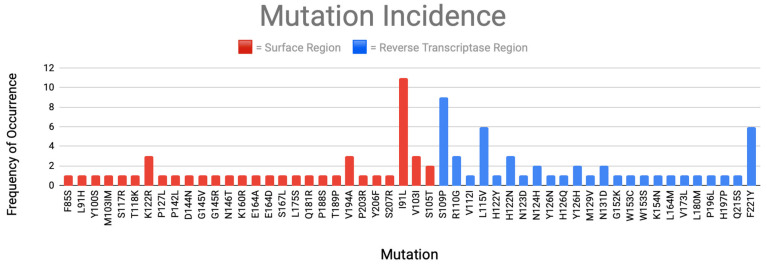
Bar graph representing prevalence of mutations in the surface region and reverse transcriptase region in people with AHD in Botswana.

Figure 2 below shows sixty-three mutations that were identified that are associated with drug resistance and immune and diagnostic escape. The most prevalent immune escape mutations were in the surface gene S207N (12/63, 19%) and A194V (9/63, 14.3%). In the reverse transcriptase region, V163I (12/63, 19%) and M129L (12/63, 19%) were highly prevalent. Two classical lamivudine-associated drug resistance mutations were observed, each occurring in one participant (L180M and V173L).

This study is the first in Botswana to report the HBsAg prevalence in people with AHD. We report the prevalence of HBsAg positivity to be 10.6%. A similar prevalence has been reported in Botswana in a cohort that had a median CD4+ T-cell count of 110 cells/µL, where the participants were ART-naïve [7]. Sarmati & Malagnino have shown that HBV prevalence is usually high in PLHIV, particularly those with a lower CD4+ T-cell count who are more at risk of developing chronic HBV infection [17]. The median age in our cohort was 37 years (IQR: 32–43), and univariate analysis revealed no association between age and HBV positivity.

## 4. Discussion

After adjusting for confounders, males and those who were ART-experienced were significantly more likely to be HBsAg-positive, as shown in Table 2. Wang et al. explained that males are more prone to HBV positivity compared to females, as the androgen pathway increases the transcription of HBV through direct binding to the androgen-responsive element sites in viral enhancer I [18,19]. The finding that ART-experienced status was associated with HBsAg positivity compared to participants that were ART-naïve is unexpected, given that HIV regimens used in Botswana mostly have an HBV-active ARV that is associated with some level of HBV seroconversion or HBsAg loss [20,21]. It is possible that the ART-experienced status in this study is associated with individuals who had lower nadir CD4+ T-cell counts compared to those that were ART-naïve, and these could have resulted in the ART-experienced PLHIV having a higher HBV reactivation, which results in them having higher HBsAg positivity [22].

Anti-HBc prevalence in our study was 50%, which is concordant with findings from other studies in Botswana [23]. Our study identified several cases of atypical HBV infection in 13.4% participants presenting with HBsAg-positive but anti-HBc-negative serology. Anti-HBc is a serological marker that normally indicates exposure to HBV; therefore, if one tests negative for anti-HBc and positive for HBsAg, it is regarded as an atypical phenotype [23,24,25]. A confirmatory serological test was done to confirm the HBsAg-reactive samples to minimize the chance this pattern reflects false-positive HBsAg results. A previous report from Botswana described this HBV phenotype and found it at a prevalence of 13.7% [23]. This HBV phenotype has been described in other settings as potentially reflecting early HBV seroconversion associated with advanced immunosuppression in PLHIV [26]. Profound depletion of CD4+ T-cells has been linked to failure to develop anti-HB antibodies, which may result in incomplete serological profiles [27]. The high prevalence of this HBV phenotype serves as a caution against HBV screening strategies that first screen for anti-HBc before testing for HBsAg and HBV DNA only in those samples that are anti-HBc-positive [28,29]. This strategy underestimates the HBV prevalence in the population.

HBV genotypes play a crucial role in HBV dynamics, endemicity, modes of transmission, and clinical outcomes [10]. In the current study, two HBV genotypes were reported, with genotype A being the most preponderant, with a prevalence of 66.7%, and genotype D accounting for only 33.3%. Our findings agree with previous studies that determined the predominant HBV genotypes in Botswana to be A and D [9]. As mentioned by Anderson et al., the sub-genotype of genotype A identified in Botswana is A1, which was found in all our genotype A samples [9]. In genotype D the sub-genotype identified was D3, which is known to be associated with occult HBV infection [30]. Phylogenetic analysis revealed that our sequences clustered with previously generated sequences from Botswana.

Sixty-three mutations were identified to be associated with drug resistance and immune and diagnostic escape. HBsAg mutants are linked to significant clinical implications such as immune escape and failure to detect HBsAg [31]. Most of the mutations identified were immune escape mutations. In the surface gene region, the most prevalent mutations were S207N and A194V. These mutations are known to be associated with failure to detect HBsAg, with A194V conferring resistance to tenofovir [32]. Additionally, we identified two lamivudine resistance-associated mutations (L180M and V173L) in the reverse transcriptase region, and these were identified in an ART-naïve patient [33]. A previous study in Botswana and one in China reported a high prevalence of lamivudine resistance-associated mutations [34,35]. This is not surprising at all, as lamivudine has a low genetic barrier to resistance, and it has been an integral part of the HIV treatment program in Botswana. The above mutations and genotyping data analysis should be understood in the context that the HBV genotyping was only successful for a limited number of samples and therefore the prevalence of the mentioned mutations might not be a true representation of their population prevalence. However, the presence of these mutations is worth mentioning to motivate future studies in more representative cohorts to explore their true prevalence.

## 5. Conclusions

There was a high prevalence of HBV in people with AHD, 10.6%, and anti-HBc, 50.0%. It is likely that in the setting of high HBV exposure, as indicated by high anti-HBc positivity, and high HBV prevalence, as CD4+ T-cells decline in PLHIV, the HBV is reactivated and the HBsAg positivity increases. This highlights the importance of HBV screening and HIV/HBV co-management in this group. Early HIV treatment initiation would also reduce HBV reactivation and potential transmission. Our study reported HBV genotypes A and D as the most prevalent, which is concordant with previous findings in Botswana. These genotypes are associated with drug resistance and immune and diagnostic escape. Further studies may explore sequencing of the whole genome to find out more about drug resistance and immune and diagnostic escape mutations in other regions of the HBV genome, as these may affect treatment.

### Limitation

The study’s main limitation is that of a lack of sufficient sample volume to screen for other HBV makers, such as HBV viral load. The ART regimen and duration of ART exposure among participants were also not available, making it challenging to link the HBV mutations with the ARVs the participants were on. Furthermore, the retrospective nature of the study resulted in the absence of important clinical data, such as liver function tests, which were not collected in the original study.

## Figures and Tables

**Table 1:** Clinical and demographic characteristics of the participants.

Characteristics	Total *n* (1097)	HBsAg-Positive (*n* = 116)	HBsAg-Negative (*n* = 981)	*p*-Value
**Sex, *n* (%)**				
Male	565 (51.5)	73 (62.9)	492 (50.2)	
Female	532 (48.5)	43 (37.1)	489 (49.6)	**0.01**
**Median Age (IQR)**	37 (IQR: 32–43)			
**Age category**				
<35 years	404 (36.8)	37 (31.9)	367 (37.4)	
≥35 years	693 (63.2)	79 (68.1)	614 (62.6)	0.24
**ART status**				
Naïve	571 (52.1)	50 (43.1)	521 (53.1)	
ART-experienced	526 (47.9)	66 (56.9)	460 (46.8)	**0.04**
**HIV viral load, *n* (%)**				
Detectable	195 (50.1)	22 (46.8)	173 (50.6)	
Suppressed	194 (49.9)	25 (53.2)	169 (49.4)	0.63
**CD4 cells/µl, *n* (%)**				
≤100	1097	116	981	0.21
**Anti-HBc status**				
Anti-HBc-positive	541	97 (86.6)	444 (45.7)	
Anti-HBc-negative	541	15 (13.4)	526 (54.1)	**<0.001**

Data are presented as No. (%) unless otherwise indicated. Abbreviations: ART, antiretroviral therapy; HBsAg, hepatitis B surface antigen; HIV, human immunodeficiency virus. Variables are highlighted in bold. Those highlighted in red show statistically significant values.

**Table 2:** Factors associated with HBsAg positivity among individuals with advanced HIV disease.

Characteristics	HBsAg Neg	HBsAg Pos	PR (95%)	*p*-Value	aPR (95%)	*p*-Value
**Sex, *n* (%)**						
Female	489 (49.6)	43 (37.1)	Ref		Ref	
Male	492 (50.2)	73 (62.9)	1.59 (1.18–2.29)	**0.01**	1.6 (1.13–2.30)	**<0.01**
**Age, years, *n* (%)**						
<35 years	367 (37.4)	37 (31.9)	Ref			
≥35 years	614 (62.6)	79 (68.1)	1.24 (0.86–1.80)	0.24	-	-
**ART status**						
Naive	521 (53.1)	50 (43.1)	Ref		Ref	
ART-experienced	460 (46.8)	66 (56.9)	1.43 (1.01–2.03)	**0.04**	1.43 (1.01–2.03)	**0.04**
**HIV viral load, *n* (%)**						
Detectable	173 (50.6)	22 (46.8)	Ref			
Suppressed	169 (49.4)	25 (53.2)	0.87 (0.51–1.50)	0.63	-	-

Data are presented as No. (%) unless otherwise indicated. Abbreviations: ART, antiretroviral therapy; HBsAg, hepatitis B surface antigen; HIV, human immunodeficiency virus; aPR, adjusted prevalence ratios. Variables are highlighted in bold. Those highlighted in red show statistically significant values.

## Data Availability

The data that was analyzed and generated in the study is not available online due to institutional policies and patient confidentiality. Data may be made accessible from corresponding author upon request, subject to approval from the institutional review board.

## Abbreviations

The following abbreviations are used in this manuscript:

HBV	Hepatitis B Virus
AHD	advanced HIV disease
PLHIV	people living with HIV
HBsAg	hepatitis B surface antigen
Anti-HBc	HBV core antibodies
aPR	Adjusted prevalence ratios
cART	combination antiretroviral therapy
CrAg	cryptococcal antigen
BEAST	Bayesian Evolutionary Analysis with the Sampling Trees software
MCMC	Markov chain Monte Carlo
MICE	Multiple imputation by chain equations
